# Thriving, not surviving: a qualitative study on barriers and strengths of first-generation medical students

**DOI:** 10.1186/s12909-026-09620-x

**Published:** 2026-06-08

**Authors:** Marco D. Boonstra, Hassan H.H. Alsadah, Netty Bos-Veneman

**Affiliations:** 1 Department of Health Sciences, Unit Education Social Medicine, University Medical Centre Groningen, Postbus 30.001, Groningen, 9700 RB The Netherlands; 2 Faculty of Medicine, University of Groningen, Groningen, The Netherlands

**Keywords:** Diversity, Equity, First-generation students, Identity formation, Hidden curriculum, Learning environment, Qualitative research

## Abstract

**Background:**

First-generation students, who do not have parents who attended university, face difficulties to succeed in academia, more stress and fatigue, less social and professional support, and less sense of belonging. They also possess unique strengths to optimize care and education. Less is known on the identity formation of first-generation students within the medical community and how they want to be supported. Therefore, in this study, we aimed to explore the experiences and the perceived barriers and strengths of first-generation medical students and their perceptions of the best strategies to optimize their experiences.

**Methods:**

We performed a qualitative study using semi-structured in-depth interviews. First-generation students in their medical bachelor were recruited by purposive and snowball sampling. Interviews were audio-recorded, transcribed, and analysed using reflexive thematic analysis. We also collected sociodemographic characteristics.

**Results:**

We interviewed 15 first-generation students. Five themes emerged from the analysis: (1) Study start, (2) The medical community, (3) Social life, (4) Connection with home, and (5) Support strategies, representing different dimensions of first-generation students’ experiences in medical school. They faced difficulties in the transition to university and experienced a mismatch between their own identity and typical traits, beliefs, and expressions of the medical community. First-generation students said to form meaningful relationships with peers but to experience a distance from more privileged students and teachers. They missed support from their family and sometimes experienced a loss of connection with them. First-generation students also expressed their strengths, such as persistence, self-trust, and understanding of society. They mentioned mentorship, peer contact, and an information platform as strategies to enhance their experiences.

**Conclusions:**

Our study underscores the need for medical education to move beyond deficit-based views of first-generation students and to actively support their identity development, social integration, and unique contributions. This can foster equity in the medical learning environment to build a more diverse and socially responsive medical workforce.

**Supplementary Information:**

The online version contains supplementary material available at 10.1186/s12909-026-09620-x.

## Background

A diverse workforce of healthcare professionals delivers better care, illustrated by improved outcomes for patients with minority or low socio-economic backgrounds [1]. Such a workforce is essential to reduce health inequalities across the globe [2], and also in the Netherlands [3]. Therefore, including and retaining students of diverse socio-economical, educational, and cultural backgrounds in medical education should be prioritized [4].

First-generation students (FGSs), defined in this study as students whose parents did not attend university, contribute to the diversity of student populations. We have chosen this definition as it brings more diverse experiences, compared to “first in family” definitions [5]. FGSs are more likely to choose primary care or family medicine as a specialty, and are more interested in working in underserved communities, such as rural areas, as United States (US) research suggests [6]. This is particularly relevant in the Dutch context, where ongoing shortages in family medicine and healthcare provision in rural areas have been identified [7]. However, in the US, FGSs are underrepresented in medical education and in science, relative to continuing-generation students, whose parents have attended higher education [6, 8]. In the Netherlands they are underrepresented as well: only 21.6% of medical students are FGSs [9].

FGSs experience both strengths and barriers within academia, as described in a scoping and systematic review, and quantitative and qualitative studies mainly conducted in the US [6, 10–14]. In educational settings, FGSs contribute with unique perspectives, shaped by their lived experiences, potentially adding different viewpoints to academic learning environments [10]. They are often motivated by responsibility towards others and by contributing to collective or family success [13]. However, FGSs often experience diminished chances to succeed in academia [11, 12], more stress and fatigue, and less social and professional support [15]. Furthermore, prevailing cultural values within universities are less compatible with those of many FGSs, possibly leading to lower performance and cultural mismatch, that in turn may lead to a lower sense of belonging [14].

Although these US studies provide important insights into the experiences of FGSs, differences between educational systems and cultural contexts may limit direct transferability to other international contexts, including the Dutch education context. For example, in the US, education has more flexible trajectories and is more costly, while the earlier tracking into educational levels in the European system can limit late entrants. Furthermore, existing studies have explored barriers and strengths experienced by FGSs, but not how these experiences relate to identity formation within medical education. Identity formation is the developmental process by which students start to think, act, and feel like a physician [16]. Identity is often defined as the set of qualities, beliefs, personality traits, appearance, and expressions that characterize a person or a group [17]. The identity of medical students is shaped by medical training, incorporating knowledge and skills from educational practices into the learner’s existing values and behaviors [16].

Furthermore, there is limited research on how FGSs can and want to be supported to enhance their experiences. Universities have launched educational initiatives to support FGSs. These initiatives often have a very specific focus, for example on university culture readiness, psychological stressors, or providing professional-social support [18–20]. Dutch universities have developed initiatives to support FGSs as well, mostly facilitating the transition from secondary school to university [21]. These support initiatives often focus on a single aspect of the transition to university rather than addressing the broad range of challenges experienced by FGSs. Whether these interventions and initiatives fulfil the needs of FGSs sufficiently or what FGSs need to optimize their study experiences is largely unknown.

FGSs’experiences of studying medicine and their support needs remain insufficiently understood. Therefore, this study aims to explore the lived experiences and the perceived barriers and strengths of FGSs who were in several phases of their bachelor of medicine, and their perceptions of the best strategies to optimize their experiences.

## Methods

### Study design

This is a qualitative study using semi-structured, in-depth interviews and reflexive thematic analysis. The COnsolidated criteria for REporting Qualitative research (COREQ) checklist was used during the development and reporting of the study [22]. Ethical approval was obtained from the Medical Ethical Review Board of the University Medical Centre Groningen by MDB and NB (project ID: 15929).

### Setting and participants

Medical students HA, AA, NM, and MB recruited participants who were in the bachelor phase of their study at the faculty of medicine, University of Groningen (UoG). The medical bachelor phase in the Netherlands comprises three years of in-faculty education. In Groningen, an English language track is provided, attracting international students and a predominantly Dutch student population. After these three years, students continue with internships during their masters.

We recruited participants via purposive and snowball sampling between April 2023 and January 2024. Participants were eligible if they did not have parents who attended higher education and were in the medical bachelor phase. In addition, our sampling intended to reach a representative sample with regards to gender, Dutch or international background, and years of studying, reflecting the characteristics of the bachelor medical student cohort at the UoG. We approached potential participants by distributing flyers throughout the medical faculty and sending out messages with flyers attached through social media platforms such as WhatsApp and Instagram. We used snowballing to recruit males who were initially underrepresented in the sample. If potential participants expressed their interest in the study, an information letter and informed consent form were sent. After giving consent, the participants were included in the study and invited for an interview.

### Data collection

The interviews took place between May 2023 and April 2024. Before the start of each interview, participants filled in a paper questionnaire to collect background characteristics. Interviews lasted between 20 and 75 min, were conducted in English, and were audio-recorded. The interviews took place in privacy in reserved rooms at the medical faculty of the UoG. The first eight interviews were conducted by HA, AA, NM, and MB in pairs. They were taking turns in leading the interview and supporting it by asking follow-up questions and monitoring topic coverage also to support their training in qualitative research. Later interviews were conducted by a single researcher (HA) once sufficient experience and familiarization with interviewing had been gained.

### Interview protocol and background questionnaire

We used an interview guide with open-ended questions (see supplementary file 1), created by HA, AA, NM, and MB, while NB and MDB provided feedback. The topics in the interview guide align with Tinto’s model of student retention, stating a combination of individual and family attributes and academic and social integration are key to ensure better study commitment and performance [23]. This model was helpful as it systematically maps how background interacts with a university’s academic and social environments, uncovering the specific institutional barriers and belonging challenges that influence students’ decisions to stay or leave. However, Tinto’s model places limited emphasis on students’ strengths, possible solutions, and experiences outside university context. Therefore, based upon literature on FGSs [6, 10] and other minoritized student groups [24, 25], we have added questions on culture, external factors (i.e. financial pressure, family responsibilities), and support to FGSs, study trajectory and study progress, social and professional support, mental wellbeing, work-life balance, culture, and family. Within these topics, we asked questions related to identity, and perceived strengths and barriers. In addition, we asked about their personal opinions about the meaning of being a FGS and advice on supporting FGSs. Questions were added to the guide after the first eight interviews to gain more in-depth experiences related to networking and sense of belonging.

We collected background characteristics (supplementary file 2) including age, gender, country of origin, native language, the highest level of education obtained by the participants’ father and mother, the country of origin of the participant’s parents, and enrollment in another study before studying medicine.

### Data handling and analysis

The audio recordings were transcribed ad verbatim, using the program F4 Transkript. The transcripts were line-by-line thematically analyzed in Atlas.ti. Analysis was initiated in parallel with ongoing data collection, and followed an iterative, inductive approach with constant reflection within the research team upon the codes and themes until data saturation was reached.

First, one transcript was analyzed independently by two pairs (AA/MB and HA/NM). One of each pair was not present during the interview to ensure an open perspective on the data. After this analysis, the two pairs discussed their coding to create a first codebook. Second, in varying pairs, four new transcripts were analyzed, while constantly adding to and refining the codebook. Third, three more interviews were analyzed with new codes emerging. After the analysis of the first eight interviews, the research team discussed the themes and the codebook in detail. They concluded that information saturation had not been reached yet and started a new cycle of recruitment and data collection. Lastly, HA and MDB analyzed seven more interviews. The research team organized the codes into code groups and derived the main themes (supplementary file 3). The themes were debated until consensus was reached.

We analyzed the background characteristics in SPSS. We used descriptives to determine the mean age and frequencies to describe other characteristics in percentages.

### Reflexivity

This study originated as a bachelor thesis conducted as a group project (HA, AA, NM, MB). After its completion, HA continued the study alongside his master’s training, while the other student researchers concluded their involvement, and thereby decided to not be an author.

The research team brought a complementary mix of perspectives. Two authors (NB and MDB) have experience in teaching and supporting medical students, and qualitative research. The other team members (HA, AA, NM, and MB) brought their student experience and were trained in qualitative methods. Two authors (NB and HA) identify as FGSs. We acknowledge that the medical students in the team shared the educational context with the participants, which may have facilitated openness during the interviews, while also introducing potential assumptions about the study context. At the same time, differences in background within the research team (e.g. first-generation and non-first-generation members, students with European and Middle Eastern backgrounds and senior researchers) were used to promote reflexivity during data collection and analysis. Throughout the analytic process, codes and themes were regularly discussed within the team to challenge individual interpretations. Despite these efforts, the researchers’ positionalities may have influenced both data collection and interpretation. For example, by shaping the focus on certain experiences or the interpretation of meaning.

## Results

### Participants

We interviewed 15 FGSs. These FGSs were in different phases of their bachelor program, and able to provide us with a variety of lived experiences. Details on background characteristics are in Table 1.

**Table 1:** Background characteristics of the 15 participants in this study

Age, mean in years, range		22 (21–25)
Gender, n (%)	*Female*	12 (80.00)
	*Male*	3 (20.00)
Country of origin, n (%)	*The Netherlands*	11 (73.33)
	*Germany*	3 (20.00)
	*Thailand*	1 ( 6.67)
Native language Dutch, n (%)	*Yes*	11 (73.33)
	*No*	4 (26.67)
Educational level father, n(%)^#^	*High*	0 (0.00)
	*Intermediate*	10 (66.67)
	*Low*	5 (33.33)
Educational level mother, n(%)^#^	*High*	0 (0.00)
	*Intermediate*	10 (66.67)
	*Low*	5 ( 33.33)
Migration background ^*^, n(%)	*Yes*	3 (20.00)
	*No*	12 (80.00)
Enrolled in an academic program before studying medicine, n(%)	*Yes*	3 (20.00)
	*No*	12 (80.00)

*n* sample size

^#^ = According to Statistics Netherlands (CBS), educational level is classified into three categories—low, intermediate and high—based on the highest completed level of education, ranging from primary and lower secondary education (low), through upper secondary and post-secondary non-tertiary education (intermediate), to higher education (high) [25, ﻿^26^]. The n for educational level for fathers and mothers is equal, but per individual student it was possible the father had low educational level and the mother mid educational level or vice versa, or that both parents had low or mid educational level

^*^ Participants were classified as having a migration background if both parents were born outside the country in which the participant grew up, regardless of the participant’s own country of birth

### Main results

Five themes emerged from our analysis: (1) Study start, (2) The medical community, (3) Social life, (4) Connection with home, and (5) Support strategies. Below, we describe the different dimensions of these themes in detail, including the supporting quotations of the participants (p).

### Theme 1: study start

Many students shared how their first steps in studying medicine were hard. They experienced difficulties and differences compared to other students in contributing to education, studying but also in the transition to their new university life. Thereby, they mentioned their persistence and trust in themselves helped to succeed.

Regarding their contribution to education, many students shared how they felt insecure about themselves and less knowledgeable compared to teachers and students with an academic upbringing. This complicated their ability to contribute to lectures or small-scale education. They said other students were better able to use the right medical wording and seemed to possess more confidence. Some FGSs came to doubt their qualities and abilities.

*Female student, native Dutch (p2)*: *‘Sometimes I felt a bit like everyone was talking in these medical terms like their parents already know everything*,* especially in the first year. Now it is not much of a problem anymore*,* and I am like “Sorry*,* I do not understand what you are saying."’*

*Male student, native Dutch (p15):* *‘At the beginning it was tough*,* because I already felt a bit out of place in medicine. During my first lecture*,* other people were very well able to answer questions about genetics and Down syndrome. And I sat there thinking “What are these people talking about?” So*,* I felt really insecure.’*

Difficulties in studying were described by most students as struggles with the high workload and time management. They mentioned how they did not know how to study the huge amount of learning material and sometimes failed tests. In relation, they told to need more time for studying and help of peers to succeed. They shared how students with higher educated parents could find this difficult to understand.

*Female student, native Dutch (p12): ‘Well*,* I feel like*,* for example*,* for me it is important to have someone to study with*,* and also have people understand that you need some more time maybe to put into your studies*,* but that’s sometimes quite difficult for me. It’s something I quite struggle with*,* that they don’t really understand that I maybe need some more time to put into my studies as they do.’*

During the transition from previous education to university, most students pointed to difficulties adapting. They faced difficulties to find information on studying and to organize themselves. Moreover, they struggled with the many changes in their personal life, for example their movement to a new city, adapting to the student life, and having to rely on yourself.

Related to the adaptation to university life, students also referred to their own persistence, motivation, and self-trust. These students mentioned how previous experiences shaped these strengths. For example, some were aware, because of their home situation, that going to university is not for everyone, leading to a high motivation to study. Others had to pave their own path to complete secondary higher education without teachers or others who believed they would succeed.

*Female student, migration background (p7):* *‘In the beginning*,* it was really hard to know what I had to do*,* I didn’t even know there were like reading materials*,* I had no idea how to find it*.* I didn’t have people around me. And then in the middle of it*,* it was hard to combine everything that I’m doing so that was also a struggle*,* but right now*,* it’s just tiring*,* it’s a lot.’*

*Female student*,* native Dutch (p3)*: *‘I think that I am confident*,* but I didn’t use to be. Other people never really believed that I had it in me to do higher education*,* but then I scored really well*,* so I did have it in me*,* but from that I learned that I should have more trust in my own ability to do things*,* because when I don’t I am kind of disadvantaging myself. I think it’s better to believe that you can do something and discover that maybe it’s not for you along the way than to already think: oh*,* it’s never going to work and then not start with it.’*

### Theme 2: the medical community

According to many students, being part of the medical community brought specific challenges. They experienced differences between their own identity and the typical traits, beliefs, and expressions of teachers, doctors, and other students.

About the medical community, most students mentioned that medicine is a competitive environment and with a strong emphasis on building up working experience and connections. They said other students could be very focused on personal achievements, while their own motivations focused, for example, more on helping patients or working collaboratively.

*Female student*,* migration background (p1)*: *‘Yes*,* it is so distant*,* and it is very…no one cares*,* you are doing it for yourself. In high school*,* if you had a fight*,* your teachers are like: what is going on? Is something going on at home. Here [at university] it is like no one is watching out for you!’*

*Male student, native Dutch (p15):* *‘The final goal of being a doctor. I would love to be a doctor*,* but I don’t like becoming a doctor*,* so that the process of medical school is really difficult. It really hardens you. It makes you competitive. It makes you bitter*,* or at least that’s what it did to me.’*

About their identity and typical traits, many FGSs mentioned that during education assertiveness and ‘being strong on your feet’ were considered important. For example, teachers or other students could behave or speak like they were better than others. This could interfere with FGSs’ own beliefs that, for example, doctors should be able to connect with all kinds of people. Some students expressed that this made them feel like an outsider, and it could be challenging to speak out if they felt something was unjust or if they disagreed. Other students considered their understanding of society or ability to connect to patients as a strength.

*Female student, international student (p4): Within medicine*,* you really notice also the lectures and so on*,* that they like sometimes talk in a certain way like oh you are doctors. Yeah*,* just a bit of an undertone that doctors are a bit better than other people in the sense which I think that also says is general mindset that*,* we as the students are always something better than other students. I think that*,* the doctors themselves should also be more aware of this and not talk like we are special because we are studying medicine!’*

*Female student, international student, migration background (p10):* *‘I have faced discrimination throughout my life*,* because I am part of a minority group*,* and different challenges because my family is not native*,* so you get really passionate proving yourself in that environment*,* because for everyone it’s so natural*,* because their parents already went to university*,* and it’s*,* their native country*,* they have it already like their system is built for them to just succeed*,* whereas the system is not built for students that have non-academic parents or migrant parents.’*

### Theme 3: social life

Most students were happy about their university social life, although they felt less connection with more privileged students and teachers. On the other hand, small-scale education and cooperating with other students broadened their social circle. Although, this was fully absent during COVID-19.

About their social life, every student said to have made friends who also act as peer supporters. These friends often were from similar socioeconomic backgrounds. Around half of the students gave examples of how they felt less connection with students with higher educated parents or parents who are physicians. Especially international students said there was a mismatch between their own culture and the typical student life. They considered other activities or studying more important than the typical student social activities and associations. One student mentioned to have joined a student association despite hesitation. It was fun and brought friends and contacts.

*Female student, migration background (p7):* *‘I think with groups*,* there are many different groups within medicine*,* You have like the bigger Dutch group that are in study associations and stuff. I don’t fit into those kind of groups*,* not at all. I have my own small friend group that I feel really comfortable in. And the doctors that lecture us*,* I used to feel very distanced to them*,* but since I am doing JSM (i.e. extracurricular research oriented education)*,* and I am working in the hospital*,* now I feel I am getting close to them.’*

*Female student, native Dutch (p3):* *‘Well*,* not that I notice this that often*,* but especially when I started going to university*,* I think it was a bit of a culture shock*,* because I wasn’t used to the student lifestyle and when I talk to my parents at home about these kind of things they didn’t really know anything about it. They would be quite shocked about how many beers people could drink on a Saturday.’*

*Male student, native Dutch (p9):* *‘I was doubting at first which student organization*,* I don’t know if this is my thing*,* they drink a lot there that’s not my thing*,* and I figured I will just try it. Now*,* I am in*,* it is really fun to be able to meet up with them. They taught me a lot about the student life. So just throwing yourself in there is probably a good thing to do*,* but I also know that some people might have more trouble with that than me*,* if you are more introverted and you don’t really like that at all*,* then it is really hard to do.’*

Some students mentioned the importance of small-scale education to create a broader social circle. They said working groups ensured contact with students and brought teachers closer. Students who started during the pandemic of COVID-19 mentioned how full-time online education diminished the possibilities of developing a social network within the community of medicine, affecting their mental health.

*Female student, international student, migration background (p10):* *‘In Germany you are not really bound to the university a lot*,* you can go to the lectures*,* and thousands of students sit with you in a lecture hall or like hundreds*,* and then you can just go home*,* because there’s little group work*,* I guess*,* and you end up not forming connections. Whereas in the Netherlands there is a lot of opportunity to talk to other students and university stuff*,* like professors.’*

*Female student, international student (p4):* *‘In the beginning with COVID*,* it was very difficult and then*,* when university started again*,* it was also a bit difficult*,* because there were some groups that had already formed. It was just a weird situation I think.’*

### Theme 4: connection with home

Most students shared their connection with parents changed since they studied. They felt supported by their parents but also experienced barriers to talk with their relatives about study experiences. They also had difficulties to find balance between study and other activities due to lacking family support.

About the support of their parents, most students shared to feel emotionally or practically supported but their parents had difficulties to understand what they were going through. Talking to them about their courses, research, and university life was challenging. They said other students were often guided by their medical doctor parents, but they had to rely on teachers, other students, or sometimes more distant family members. Some students did not share much with their parents about studying, because it did not bring them further. Other FGSs’ parents did not understand their study decisions or even disapproved.

*Female student, migrant background (p1):* *‘My mom*,* she wasn’t per se the happiest*,* which sounds very weird*,* when I started studying medicine*,* because she wasn’t fond of me moving away as well*,* because I’m the first*,* let alone like person*,* there’s also a big difference between girls and boys in my culture. I’m the first family member in a lot of generations that ever moved away from home for studies*,* so that is a really big deal.’*

*Female student, international student (p13):* *‘My friends are doing internships and maybe they are writing papers and their parents can like help*,* like “oh you should do this*,* you should contact this person*,* maybe help read their papers”. That’s something my parents can’t really help me with*,* or especially with like nepotism in the medical world. I think it helps when you have parents that have gone through higher education.’*

Related, some students shared how their ‘moving up the educational ladder’ could make them an outsider in their family. For example, they felt their family had other perspectives on societal issues or saw them as overachievers. At the same time, they felt their social backgrounds could help them in the future by having unique qualities, such as persistence and a broad perspective on society.

*Female student, native Dutch (p8):* *‘I think at the end of high school*,* that’s when a teacher told me. You do know that it is more difficult for you to do a subject than for other people because*,* for example*,* their conversations at the dinner table are different than the conversations I had. Yeah. I mean*,* my parents would still start a discussion about the zwarte piet [i.e. zwarte piet is/was a blackface character in the Dutch Sinterklaas celebration - the discussion was about if this is a racist stereotype*,* and more and more piet is replaced by a more culturally sensitive version]. Yeah. And I just get really mad about that. And other people were on another level*,* they had discussions. So*,* yeah*,* I think I realized that I have further to come from. But that also means that you deserve even more to be there. Yeah*,* they should be even prouder than you are there.’*

*Female student, native Dutch (p3):* *‘I came from [an upbringing] like “you have to kind of work hard*,* we are all the same*,* no one is better than anyone else”. So that mentality*,* so I think it’s also sometimes a bit of an advantage*,* because now I work with like*,* for example*,* patients and I really kind of know what it is like to live in a certain environment*,* because for example my grandparents are very similar to them.’*

With regard to balancing study with other activities, many students said they had to work or take care of family, making studying more challenging. Taking care of family could also imply the switching of roles, for example when students helped their family out in administrative tasks. Half of students shared working was necessary because financial support from their parents or government was insufficient.

*Female student, native Dutch (p2):* *‘My parents tried to support me*,* but they don’t make that much money*,* so they can’t really like pay for my study or anything*,* so just like… I am taking a loan right now*,* so right now I don’t have any worries*,* but I need to pay it all back. So I have some friends in high school and their parents were like highly educated and they had a lot of money as well*,* so they were like buying an apartment for their kids to live in the city and obviously my parents didn’t have that kind of… financial ability’*.

### Theme 5: support strategies

Most students reflected upon available support for students in medicine that did not always meet their needs. They suggested multiple support strategies.

FGSs mentioned several available support options, such as study advisors, teachers from small-scale education, WhatsApp groups of students, and websites. These options were useful for practical questions about study delay, tests, or the curriculum. However, students did not always have the courage to ask questions, or the teachers were not always approachable. Moreover, these support options did not provide help in other situations related to being a FGS, such as being introduced to studying at university, helping in building a network, meeting peers, contributing to personal development, connecting with family, and organizing housing or finances.

*Female student, native Dutch (p12):* *‘Well*,* I think*,* mostly*,* having someone to go to and talk about it*,* because I don’t see how you can integrate it in the studies. We have a study advisor*,* but I still feel it is more about the practical stuff of the studies*,* and I wouldn’t really know who to go to if it was more about the social side*,* or that kind of stuff.’*

*Female student, international student, migrant background (p10):* *‘I think all my tutors were super nice*,* and they were still like students as well*,* there was still the feeling that they are not way too far from me*,* age wise as well*,* but I guess also the way that they do not look too different to us and they would just look friendly*,* so when I had issues with you know*,* organizational things or questions*,* I would turn to them*,* I would ask them over WhatsApp*,* what to do and how to handle this*,* and they were always super helpful.’*

Students suggested other strategies to improve the experiences of FGSs. To overcome the disadvantaged start students advised to set up an information platform and introduction program for new students. They pointed to mentorship, role models, and peer contact, also in small scale education, as tools to help FGSs to build a professional and social network, and to develop themselves regarding insecurity and professional identity. Some students valued to have information for parents although others did not find that necessary. One student thought a better faculty policy on inclusive teaching and equity was needed.

*Female student*,* international student (p4)*: '*I think maybe something you know, like a website where they explain the support and where to get it and then just*,* you know*,* send out an e-mail that people then know*,* oh*,* if I have these issues*,* I can look it up again. I think that might be like a middle ground.* *Female student*,* international student (p4) [later in the interview]*: *I think it would make a lot of sense to have a lecture specifically for parents*,* and I think maybe a video or something that they would get just to explain maybe a bit more how university works. Yeah*,* I think maybe that’s a possibility.'*

*Interviewer*: '*Um*,* and would you also like more help from others*,* let’s say supervisors*,* mentor*,* or coach? There is also the student advisor.* *Female student*,* native Dutch (p12)*: *Yeah*,* I think I would like someone where you just have to talk to every three months or so*,* and you can just talk about how studying is going*,* and not only about the subjects*,* but just how are you dealing with studying*,* and how is it going with you*,* do you have anyone to talk to*,* or that kind of stuff.'*

## Discussion

Our study disclosed the experiences and perceived barriers and strengths of FGSs who were in their bachelor of medicine, and their perceptions of the best strategies to optimize their experiences. Theme 1 and 2 show that FGSs experience difficulties in their transition to university and differences between their own identity and the traits, beliefs, and expressions of the medical community. Theme 3 and 4 reveal that FGSs have a satisfying social life within university with peers. However, many experience distance from more privileged students and teachers, and staying connected to family is challenging. Within all themes, FGSs expressed various strengths, such as persistence, self-trust, understanding of society, and being able to connect to people with lower socioeconomic positions. Theme 5 indicates that mentorship, peer contact, and an information platform are the most promising tools to enhance the experiences of FGSs.

Our findings align with previous literature suggesting that the transition to university is particularly challenging for FGSs [6]. Large-scale and qualitative studies have shown that, compared to continuing-generation students, FGSs report lower use of institutional support, higher levels of stress [27] and more limited access to academic and social resources [28]. These findings can be further understood through Tinto’s model of student integration [23]. Our results illustrate that FGSs experience challenges in different domains of this model: difficulties in navigating academic expectations during the study start reflecting limited academic integration, while feelings of distance from peers, teachers, and the medical culture point to constrained social integration. However, our study adds to this by emphasizing the role of identity-related tensions between students’ personal backgrounds and the dominant culture of the medical community. Comparable tensions have been described among students from lower socioeconomic, other cultural and queer backgrounds. In these students, mismatches between personal and professional identities affect learning experiences, sense of belonging, and professional development [29, 30]. In medical education, professional development programs often prioritize competency acquisition while paying limited attention to personal identity as a starting point for professional identity formation [24]. As a result, FGSs are frequently conceptualized in terms of deficits or risks related to academic performance. Ives et al. propose a more strengths-based perspective, in which students’ lived experiences enrich learning and support personal and professional growth [10]. Relatedly, our findings suggest that difficulties in transition are not solely related to resources, but also to deeper processes of identity negotiation within medical training and underscore the need for pedagogical approaches enabling students to develop a professional identity that aligns with their own values and experiences [31].

Our results on social life and connection with family are concordant with other studies reporting that FGSs and other minority group students have feelings of exclusion, discomfort or intimidation [27, 32], and experience challenges to be supported by and stay connected to family [20, 33]. The results from our study add that FGSs feel happy in a group of students from similar backgrounds, but feel more distance from continuing-generation students and teachers. Interview studies among 60 US students show how feelings of exclusion and lesser sense of belonging bring identity related conflicts. For example, FGSs conceal where they grew up, while they feel pressure to reconcile their new identity with family expectations as well [34]. The hidden curriculum within medical education influences the experiences of FGSs more than continuing-generation students, as they are less familiar with the expected social norms, behaviours, and values, communicated implicitly via university structures, routines, and social interactions. From a Tinto perspective [23], such implicit norms, values, and behaviours may hinder full social integration, particularly when students lack prior exposure to these expectations, thereby reinforcing feelings of marginalisation despite formal participation in the educational setting [13, 14, 33]. To optimize experiences of FGSs, we advocate to reveal the hidden curriculum and to build inclusive communities together with students, teachers, and policy makers.

Within the different themes of our results, students mention several strengths of FGSs, such as motivation, persistence, and being able to understand patients from lower socioeconomic status. Our results confirm that FGSs express to have learned to be persistent [13], and expect to possess more empathy and culturally sensitivity, especially towards underserved patient populations [6, 35]. These characteristics relate to the competencies of leadership, health advocacy, and communication, that are much needed to face nowadays societal challenges, such as increasing health disparities, ageing, and lifestyle-related chronic diseases. In line, Romero et al. suggest to increase outreach and support of FGSs, leading to a more diverse workforce, better equipped to provide culturally sensitive care and to reduce health disparities [12]. We embrace this suggestion and, in addition, want to emphasize the importance to support FGSs over multiple years to further develop their strengths and talents.

In our study, FGSs suggest various strategies to improve their experiences. Mentorship, peer contact, and an information platform were most often mentioned. Other studies have reported similar strategies to provide guidance, emotional and practical support to FGSs, and to build communities as a buffer against feelings of isolation and marginalization. Studies in the US provide some evidence on their effectiveness. For example, mentorship was effective to optimize graduation from high-schools and university enrolment, to progress within university, and foster feelings of belonging and self-efficacy [36]. Another study among minority students, reported that individual mentoring by a teacher enhanced career planning, feeling connected, and navigating academic and social challenges [37]. Thus, these strategies are promising to improve the experiences of FGSs in medicine.

### Strengths and limitations

Our study has several strengths. A first strength is the embedding of the FGSs’ perspective in all phases of the research through close cooperation between students and experienced researchers. This helped to strengthen our methods, for example by linking theory to practical experiences in the interview manual. Second, all interviews were conducted by student researchers, who were in the same study phase as the participants, fostering an open environment with little power dynamics although, first-year students might look up to third-year students. A third strength is the composition of the sample, representative of the student population in Groningen with various backgrounds. This provides a variety of perspectives on experiences of FGSs.

Our study also has limitations. First, the interviews were done in English to foster equity in participation. The student researchers and participants were from different countries. English is not their native language, and its use might have influenced the depth of the interviews. Although, we expect this influence is marginal, as the student researchers took the IELTS (International English Language Testing System) test and participants were invited to answer in Dutch if they struggled in speaking English. Second, the fact that two researchers identify themselves as FGSs, may have increased sensitivity to themes such as belonging and social expectations, and may have influenced our snowball sampling, when recruiting from personal networks. Third, we did not ask participants to check the summary of their interview, a triangulation method, to heighten the credibility of our study. However, we expect our collaborative and iterative approach to have prevented misinterpretation.

### Implications

Our results lead to implications through which universities may improve FGSs’ experiences. First, we suggest incorporating mentor groups into the curricula of studies in medicine. In these groups, students can discuss the role of their background in learning and the development of their professional identities. The groups can also help to discuss personal experiences and strategies to counteract negative experiences. Second, faculties should actively work towards building an inclusive medical community by making implicit norms and expectations more explicit. This can be achieved by training teachers, supervisors, and study advisors to use inclusive language, to explicitly explain unwritten rules (e.g. around networking or career planning), and by proactively checking in with students who may be less likely to seek support. Third, universities should invest in targeted support programs to facilitate FGSs’ transition into and through medical education, such as tailored introduction activities and accessible information platforms. In addition, providing outreach activities in secondary education and continued support during clinical training and early career stages may help FGSs to build networks, navigate institutional structures, and further develop their strengths, ultimately contributing to a more diverse and socially responsive medical workforce.

Our study also holds implications for research. Longitudinal studies could provide insight into how FGSs’ experiences evolve over time, not only while studying at the university, but also while going through primary and secondary school or during their careers as doctors. More specific, research is needed on the strengths of FGSs and how these can be empowered throughout the life-course. Future research should also explore intersectionality — for instance, how experiences differ by gender, migration background, or socioeconomic status - and examine how institutional cultures themselves are reproduced or disrupted by FGSs. Last, research should develop and evaluate the suggested intervention strategies to learn about their strengths, weaknesses and effectiveness.

## Conclusions

In conclusion, our study underscores the need for medical education to move beyond deficit-based views of FGSs and to actively support their identity development, social integration, and unique contributions. This can foster equity in the medical learning environment to build a more diverse and socially responsive medical workforce.

## Supplementary Information

Supplementary Material 1.

## Data Availability

The data that support the findings of this study are available from the University Medical Center Groningen, Department of Health Sciences, Unit education Social Medicine, but privacy restrictions apply to the availability of these data. Data are however available from the authors upon reasonable request and with permission of the authors.

## Abbreviations

FGSs: First-generation students
UoG: University of Groningen
