# Supplementary material for: Thriving, not surviving: a qualitative study on barriers and strengths of first-generation medical students
Source: BMC Med Educ. 2026 Jun 8;26:1202. doi: 10.1186/s12909-026-09620-x (PMC13393850; doi:10.1186/s12909-026-09620-x)
Supplement: Supplementary file 1 — Supplementary Material 1. [file 12909_2026_9620_MOESM1_ESM.docx]

# Supplementary files

## Supplementary file 1: Interview protocol

| **Introduction** | We would like to thank you for participating in this study. Our names are …… & ……… . and we are doing research together with three other colleagues. The research is about the life and study experiences that first generation medical students face and how to improve them. We would very much like to listen to your story about what you experienced during your study. This way, we might be able to help and support future first-generation students. This interview will, expectedly, take 50-60 minutes. We will record your answers by audio during the interview. We do this to be able to go back to it and listen to what you said. We will store the recording in a secured computer to protect your privacy. During the interview, feel free to elaborate as much as you want and feel comfortable with. If at any time you feel uncomfortable, please inform us. Is this clear? Do you have questions? |
| --- | --- |
| **Opening question** | We will start recording from this moment on, is it okay with you?  Before we start officially, we would like to know more about you. Could you introduce yourself to us?  **To start with, could you explain in a few sentences why you decided to participate in this study?** |
| **Individual attributes, drives / values**  **Study progress**  **Support**  **Individual attributes / prior qualifications** | **Why did you decide to study medicine ?**   - Is studying medicine how you expected it would be? - Why/why not? (positive vs negative experiences/specific events) - How did you feel during those experiences?   **In general, how is your study of medicine going?**   - How is your progress? - If things are going not so well: do you have explanations?   **Thinking as broad as you can, what did you consider complicated during your studies?**   - What parts of the study were or are difficult for you? - Why do you think you have difficulties with this part? - How is planning and organizing everything in your studies going?   **If you encountered difficulties during your studies, who did you turn to at the university?**   - What kind of questions did they help you with? - How helpful was the support you received? - How easy/difficult was it to find support?   **What competencies do you think you need to develop to successfully progress in medicine?**   - What past experiences did you bring that help you in medicine? - Do you think being a first-generation student helped in any way? How? |
| **Family / social network and social integration / sense of belonging** | **What is the role of your social network in supporting your studies?**   - How are/were you supported by your family exactly (practical guidance, emotional support, other)? - Have you experienced any major life events during your studies? (death, divorce (parents), pregnancy, marriage etc.)   - If yes: how did this effect your studies?   **To what extent do you feel part of the group of students studying medicine and the doctors that play a role in teaching?**   - Can you explain why you feel or do not feel part of it? - What challenges did you face when you aimed to connect with fellow students or doctors? - Were you able to build a professional network (students/doctors etc.)? |
| **Mental well-being** | **How is/was your mental wellbeing during your studies?**   - Considered good 🡪 how did you maintain good mental health? - Considered bad 🡪 How did you deal with this? - Do you feel confident you will succeed in your studies? - In what way is this influenced by being a first-generation student? |
| **External factors** | **How is your work-life balance?**   - How do you deal with the workload? - Do you have sufficient time for hobbies? - Do you have a job to support your study?   - If yes: how do you balance your job with your studies? - Have you faced any financial problems due to studying medicine?   - If so: what did you do to overcome them? - In what way is this influenced by being a first-generation student? |
| **Culture and academic integration** | **Considering you are a first-generation student. What was it like to enter the system of higher education?**   - Did your background influence your experience in higher education? (language, religion, customs)   - If yes: how? - To what extent do you think your own culture or background is different from the medical community, can you elaborate? - What strengths do you bring because of your background to the medical community or later, as future doctor? |
| **Personal identity** | **We asked you a few questions about different themes related to your studies. For you, which difficulties do you feel like are influenced most by being a FGSS?**   - For the positive aspects you mentioned: in what way were these influenced by being a FGSS? - What does being a FGSS mean to you?   **Were there things that positively influenced your studying experience?**   - In what way did this help you? |
| **Suggestions and Advice** | **Do you have any advice for other FGSS currently studying?**   - Do you have any suggestions for universities/family? - How can FGSS best be supported? |
| **Word of appreciation for participating** | We would like to thank you in the end for dedicating your time to share your experiences with us and help us gain insights that would hopefully support and empower FGSS during their studies in the future.  Before we end this interview, do you have any questions or remarks that you would like to direct to us?  Do you have any other remarks or advice that you would like to share? |

## Supplementary file 2: Background characteristics questionnaire

Participant’s number:
Date of the interview:

Please fill in the following questions:

| Year of birth |  | | |
| --- | --- | --- | --- |
| Gender | Male | Female | Other: |
| Country of origin |  | | |
| Are you a native Dutch speaker? | Yes | | No |
| Did you enroll in another academic program before studying medicine? | Yes | | No |
| I have parents that have attended higher education (university or university of applied science) | Yes | | No |
| Highest education of both parents: (mention low, intermediate or high, and if necessary explain) | The father: | | The mother: |
| Country of origin of both parents: | The father: | | The mother: |

## Supplementary file 3: Code book

| **Name** | **Definition** | **Theme** |
| --- | --- | --- |
| Academic pressure | The perceived pressure to perform academically | Theme I: Study start |
| Workload | The perception of the amount of work necessary to succesfully progress in the study | Theme I: Study start |
| Time management | The way the participant manages their time | Theme I: Study start |
| Language barriers | Language barriers perceived by the participant | Theme I: Study start |
| Study progress | The way the participants are progressing over time during their study | Theme I: Study start |
| Adapting to changes | To what extent the participants experience difficulties to adapt to changes in their personal life or the setting of higher education | Theme I: Study start |
| Stress | The participants' experience of feelings of stress | Theme I: Study start |
| Coping mechanism | The way the participants deal with stressful factors or barriers | Theme I: Study start |
| Concentrating | The participants ability to concentrate on study material | Theme I: Study start |
| Level of confidence | The lack of confidence that the participant is feeling | Theme I: Study start |
| Competency development | Skills that are considered important to develop successfully in medicine | Theme I: Study start |
| Educational moments | The extent to which participants feel they can contribute to educational moments | Theme I: Study start |
| Knowledge | The extent to which participants feel they possess sufficient knowledge to study medicine | Theme I: Study start |
| Motivation / Persistance | Going through the study by facing problems and showing persistance to overcome them | Theme I: Study start |
| Problem solver | Experiences in which the participants had to solve study problems for themselves | Theme I: Study start |
| Comparison with others | Participants comparing their knowledge and skills with those of others | Theme I: Study start |
| Being selfmade | Creating / using the own strengths as an identity asset | Theme I: Study start |
| Advise: assertiveness | Participants advising other first-generation students to be assertive during their studies | Theme I: Study start |
| Advise: finding balance | Participants advising other first-generation students to not stress too much, find balance | Theme I: Study start |
| Advise: be proud | Participants advising other first-generation students: find pride in being the first to go to university | Theme I: Study start |
| Perception of doctors | Perceptions of the participants on doctors educating them | Theme II: The medical community |
| Perception of other students | Perceptions of the participants on students who are not first-generation | Theme II: The medical community |
| Beliefs / opinions about themselves | Typical beliefs of opinions of people within the medical community about themselves | Theme II: The medical community |
| Beliefs / opinions about others | Typical beliefs of opinions of people withing the medical community of others in society | Theme II: The medical community |
| Participants beliefs / opinion | Participants' beliefs / opinion about society / people of low SES | Theme II: The medical community |
| Change of perspective | How being part of the medical community sometimes changes the participants' perspective | Theme II: The medical community |
| Differences FGS and community | Expressions that illustrate how the participants feel different from the medical community | Theme II: The medical community |
| Role models | Persons that are within the doctor community and serve as a role model for the student | Theme II: The medical community |
| Discrimination | Examples of discrimination perceived by the participants in the educational setting | Theme II: The medical community |
| Doing medical jobs / internships | The influence of doing medical jobs or internships on shaping your identity as a doctor | Theme II: The medical community |
| Being selfmade | Creating / using the own strengths as an identity asset | Theme II: The medical community |
| Traits of the medical community | Examples of typical traits of doctors or students within the medical community | Theme II: The medical community |
| Student-teacher relationship | The relationship between participants and their teachers | Theme II: The medical community |
| Drive / motivation | What drives the student to study medicine or to learn content of the studies | Theme II: The medical community |
| Professional network | The participant's professional network and contacts within the medical field. | Theme II: The medical community |
| Advice: strong mentality | Participants advising other first-generation students: create a strong mentality / believe in yourself | Theme II: The medical community |
| Advise: be proud | Participants advising other first-generation students: find pride in being the first to go to university | Theme II: The medical community |
| Sense of belonging | The sense of belonging perceived by the participant due to shared experiences | Theme III: Social life |
| Difficulty connecting on social level | The participant experiences difficulties connecting on social level with their peers | Theme III: Social life |
| Free time | The way the participant spends their free time | Theme III: Social life |
| Peer support | The support the participant receives from other medical students | Theme III: Social life |
| Grouping | The grouping of people with similar backgrounds | Theme III: Social life |
| Cultural differences / mismatch | The differences resulting from culture / backgrounds perceived by the participants | Theme III: Social life |
| Student association and activities | Experiences related to being part of a student association and activities within the community | Theme III: Social life |
| Experiences in (previous) education | To what extent the schooling system can help to foster belonging | Theme III: Social life |
| Free time | The way the participant spends their free time | Theme III: Social life |
| Influence of Covid-19 | The influence of the pandemic that happened in 2019 caused by SARS-virus | Theme III: Social life |
| Social network | The participant's social network within and outside university | Theme III: Social life |
| Advice: active participation | Participants advising other first-generation students: become active within the community | Theme III: Social life |
| Advice: build a social network | Participants advising other first-generation students: ways to build a social network | Theme III: Social life |
| Financial issues | Financial issues the participant is facing while studying medicine | Theme IV: Connection with home |
| Financial support | The support that the participants get whether it is from a job, the government or their social network | Theme IV: Connection with home |
| Practical support | To what extent the family network around the participant can support practically | Theme IV: Connection with home |
| Understanding from family members | The perceived understanding from the family of the participant about studying medicine | Theme IV: Connection with home |
| Family's experience | The experience in higher education of the participant's family broadly (so beyond parents) | Theme IV: Connection with home |
| Emotional support | The emotional support perceived by the participant from their family members | Theme IV: Connection with home |
| Other family being the supporter | When another family member takes over the role of the parents in supporting studying medicine | Theme IV: Connection with home |
| Conflict | Examples of how students experienced conflict with their families related to becoming educated | Theme IV: Connection with home |
| Switched roles | When the student is taking responsible roles for the parents or other family members | Theme IV: Connection with home |
| Exclusion of family members | The participant is excluding family members in their experience of medical school | Theme IV: Connection with home |
| Family's expectations | The participant's perception on their family's expectations | Theme IV: Connection with home |
| Combining work/personal life | How the participant combines their personal life (family, friends, hobbies) and work life (study, job) | Theme IV: Connection with home |
| Views on being higher educated | Views of people who did not attend higher education about people who did attend higher education | Theme IV: Connection with home |
| The role of background | How participants felt their background could play role in their professional journey | Theme IV: Connection with home |
| Advice: family | Participants advising other first-generation students how to include or deal with family | Theme IV: Connection with home |
| Access to support | The accessibility of educational support | Theme V: Support strategies |
| Guidance | To what extent participants feel guided by the university | Theme V: Support strategies |
| Quality of educational support | The quality of educational support in relation to being a first-generation student | Theme V: Support strategies |
| Seeking educational support | The participant reaching out for educational support | Theme V: Support strategies |
| Available support options | The participant sharing about available support options within university | Theme V: Support strategies |
| Information platform | Advice to build an information platform to support first-generation students | Theme V: Support strategies |
| Introduction program | Advice to develop an information platform to support first-generation students | Theme V: Support strategies |
| Mentoring / small-scale education | Advice to use mentoring or small-scale education to support first-generation students | Theme V: Support strategies |
| Other advices for universities | Other advices for the university on how to support first-generation students better | Theme V: Support strategies |
| Advice: Ask for advice | Participants advising other first-generation students on how to seek advice or guidance | Theme V: Support strategies |
